# A Review of the Properties of Clinically Evaluated Plant-Derived Agents in the Treatment of Respiratory Infections

**DOI:** 10.3390/nu18101534

**Published:** 2026-05-12

**Authors:** Alexandra S. Alexandrova, Vasil S. Boyanov, Liliya Y. Boyanova, Raina T. Gergova

**Affiliations:** Department of Medical Microbiology, Medical Faculty, Medical University of Sofia, Zdrave Str. 2, 1431 Sofia, Bulgaria; v.boyanov@medfac.mu-sofia.bg (V.S.B.); liliya.boianova@medfac.mu-sofia.bg (L.Y.B.); r.gergova@medfac.mu-sofia.bg (R.T.G.)

**Keywords:** *Echinacea* spp., *Pelargonium sidoides*, *Hedera helix*, *Thymus vulgaris*, *Althaea officinalis*, *Sambucus nigra*, *Zingiber officinale*, *Curcuma longa*, upper respiratory tract infections, lower respiratory tract infections

## Abstract

**Background**: The use of plant-derived agents is a common approach in integrative care for respiratory conditions. However, the evidence of clinical trials has not yet been comprehensively presented. **Aim**: To summarize the antibacterial, antiviral, immunomodulatory, antioxidant, and expectorant properties of *Echinacea* spp., *Pelargonium sidoides*, *Hedera helix*, *Thymus vulgaris*, *Althaea officinalis*, *Sambucus nigra*, *Zingiber officinale*, and *Curcuma longa*, and to evaluate the evidence level from clinical trials (CTs) involving these agents in patients with respiratory tract infections (RTIs). **Methods**: We conducted a literature search using the PubMed database focusing on clinical studies of plant-derived agents in upper and lower RTIs. PRISMA-based reporting elements were used only as a guiding tool for comprehensibility of the literature search (Reporting Items for Systematic Reviews and Meta-Analyses guidelines). **Results**: A summary and structured overview of the properties of these most-cited plant-derived agents in the literature, in the context of RTIs, was provided. A total of 94 reports met the eligibility criteria and were included in our review. Of these, 66 reported randomized and placebo-controlled trials investigating the efficacy and tolerability of these adjuncts in patients with RTIs. The non-randomized and uncontrolled trials were 22. Sufficient evidence to be regarded as an appropriate treatment to reduce the severity and duration of RTIs was found for all discussed plant-derived agents. Robust evidence available was found for *Echinacea* spp., *Pelargonium sidoides*, *Sambucus nigra*, *Curcuma longa* and *Zingiber officinale*. **Conclusions**: Regarding other plant-derived agents reported in the traditional medicine for the treatment of RTIs, further research is needed to clarify the evidence gaps.

## 1. Introduction

Upper and lower respiratory tract infections (URTIs and LRTIs) are prevalent health concerns globally, accounting for a significant burden of illness and healthcare resources. URTIs include conditions like nasopharyngitis, sinusitis, tonsillopharyngitis, adenoiditis, and laryngitis, whereas LRTIs encompass more serious infections such as bronchitis, bronchiolitis and pneumonia [1]. The standard medical management, which comprises agents for supportive care, such as hydration and rest, along with antimicrobial therapy when necessary, is crucial for effective treatment. The widespread and often inappropriate use of antimicrobials in human medicine, veterinary practice, agriculture, and self-medication has accelerated the emergence of resistant microorganisms [2,3,4,5,6]. As a result, common infections are becoming harder to treat, leading to prolonged illness, increased healthcare costs, higher mortality, and a growing burden on healthcare systems worldwide [7,8].

In response to the escalating antimicrobial resistance crisis, there is an urgent need not only for new antimicrobial drugs but also for alternative and complementary therapeutic strategies that can reduce reliance on antibiotics. One promising approach is the exploration of plant-based agents as adjuvant therapies. Medicinal plants have been essential in traditional medicine for centuries, due to the various bioactive compounds that provide significant health benefits, including potent antimicrobial and antiviral properties [9,10,11].

Some phytochemicals can weaken bacterial defences, inhibit biofilm formation, or interfere with quorum sensing [12,13,14,15]. Others support the host immune response or alleviate symptoms, potentially reducing the need for antibiotic use in self-limiting infections such as uncomplicated respiratory tract infections. A wide range of plant-based products has a long-standing history of use and is gaining significant attention in research for the effective management of RTIs. These products are recognized for their powerful anti-inflammatory, antitussive, expectorant, demulcent, and immunomodulatory properties [16,17,18,19]. However, their use should remain adjunctive to standard medical care. Clinical decisions should be based on the quality of available evidence, safety considerations, and the individual characteristics of each patient [20,21,22].

The plant-derived agents are increasingly studied for their potential to enhance treatment outcomes when used alongside conventional therapy. These natural remedies serve as supplementary therapies to help relieve symptoms, enhance immune function, and promote recovery from respiratory infections. The use of evidence-based herbal products for symptomatic relief can help reduce inappropriate antibiotic prescribing and self-medication. In many countries, medicinal plants are widely recognized and serve as a primary form of healthcare. Over 80% of people in low- and middle-income countries rely on these plants for their health needs [23,24]. In rural India areas, traditional plant-based remedies are used by ~70% of people for general health, including respiratory conditions [25]. In Germany ~61.8% of people with RTIs used herbal products or dietary supplements [26]. Bulgaria was found to rank 3rd globally by the number of medicinal plant species reported for symptom relief of respiratory disease, after Nepal and Thailand [27]. The plant-derived agents are popular due to their availability, accessibility, affordability, and the perception that they are safer than modern medicine. The effectiveness of medicinal plants is attributed to their bioactive compounds, such as essential oils, polyphenols, flavonoids, saponins, and alkaloids, which have been reported to relieve symptoms of acute URTIs such as tonsillitis, tonsillopharyngitis, and rhinitis [28,29,30,31] and acute LRTIs, such as acute bronchitis and acute cough [32,33].

**Aim.** This review highlights the properties of plant-derived agents, subjects of clinical trials, which have been evaluated for symptomatic relief in upper and lower RTIs.

## 2. Materials and Methods

### Search Strategy, Eligibility Criteria, and Selection

**Figure 1:**
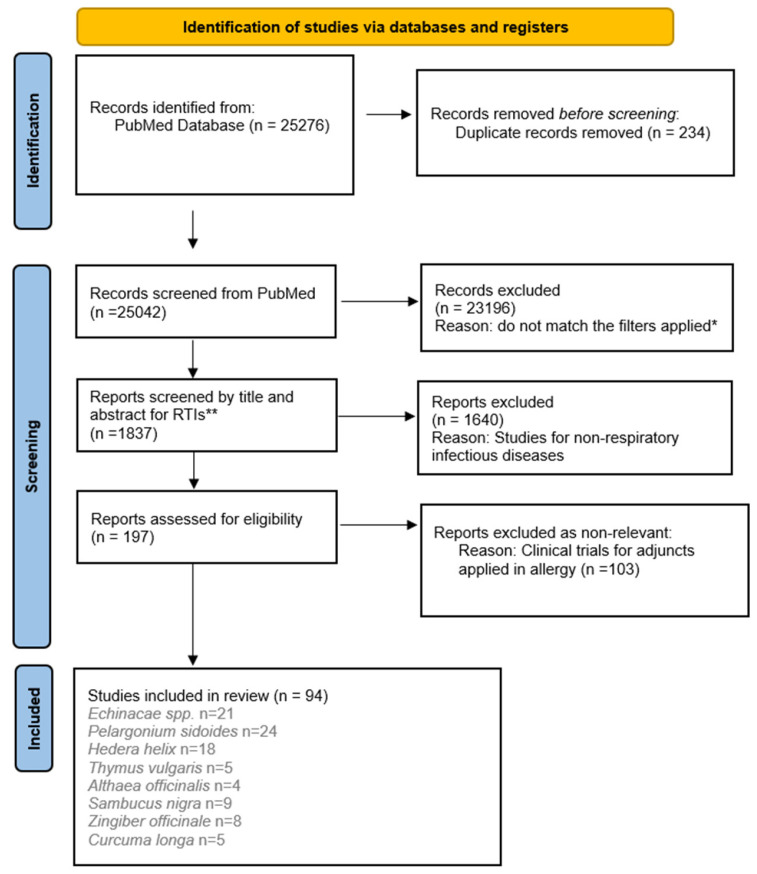
Elements of PRISMA diagram of studies relating to Plant—derived agents in clinical trials 2000–2025 used as supplementary therapy for respiratory tract infections. * Applied filters: Adaptive Clinical Trial, Clinical Study, Clinical Trial, Clinical Trial Protocol, Comparative Study, Controlled Clinical Trial, Multicenter Study, Pragmatic Clinical Trial, Randomized Controlled Trial. ** RTIs—respiratory tract infections.

We conducted a literature search using the PubMed database. For improved clarity, we utilized PRISMA—based reporting elements as a guiding tool to promote transparency, rather than strictly following specific methodological requirements (Figure 1). By incorporating these components, we aimed to enhance the clarity and comprehensibility of our literature search, selection, and reporting processes. The search strategy was constructed using inclusion and exclusion criteria. The inclusion criteria for this review were established though a search strategy combining various keywords including (“respiratory infection” OR “respiratory” OR “inflammation” OR “cough”, “flu”, “cold”, “sinuitis”, “tonsillitis”, “pharyngitis”, “laryngitis”, “bronchitis”, “pneumonia” AND (“the name of the plants selected from a screening for most cited plants used as supplementary agents in the therapy of RTIs”) in PubMed. The plant names have been checked with “World Flora Online” (on www.worldfloraonline.org, version 2.0, accessed on 22 February 2026).

The search was conducted by one of the researchers (ASA). The following eligibility criteria were established for inclusion in this review:

(a) studies investigating the effect of the following plants for medicinal purposes: *Echinacae* spp., *Pelargonium sidoides*, *Hedera helix*, *Thymus vulgaris*, *Althaea officinalis*, *Sambucus nigra*, *Zingiber officinale*, and *Curcuma longa*; (b) in vitro designs, in vivo (animal models), and randomized and non-randomized trials were accepted. The filters applied during the search were: Adaptive Clinical Trial, Clinical Study, Clinical Trial, Clinical Trial Protocol, Comparative Study, Controlled Clinical Trial, Multicenter Study, Pragmatic Clinical Trial, Randomized Controlled Trial.

The exclusion criteria encompassed duplicate publication, studies addressing a natural compound but not related to RTIs, research on RTIs unrelated to natural compounds, and articles referring to herbal remedies from traditional medicine that had not been scientifically evaluated for RTIs. PhD theses were not searched since we did not perform quality assessment or risk of bias analyses of the included studies. The exclusion criteria comprised non-research articles, systematic reviews, non-systematic reviews, and in silico studies.

We placed a restriction on publication dates, selecting only articles published between January 2000 and December 2025.

The reference lists of original studies were manually searched to identify articles that could have been missed during the initial online search.

The titles and abstracts were screened using Rayyan software (https://www.rayyan.ai/, version 1.7, accessed on 22 February 2026) by two reviewers (ASA and VSB) to apply the eligibility criteria. The software made it possible to classify each reference as included, excluded, or uncertain. All papers included by at least one reviewer or whose inclusion was uncertain were preliminarily accepted and left for full-text review.

Duplicate studies were removed using a reference manager (Mendeley Desktop version 1.19.4 ©2008–2019 Mendeley Ltd. Kidlington, Oxford, UK) accessed on 5 March 2026.

Four reviewers (ASA, VSB, LYB and RTG) evaluated the full papers, and a decision was made for each one. The search process was finished on 22 March 2026.

The following information was extracted from the included papers: first author, year of publication, country in which the study was conducted, type of study, and evidence level.

A brief summary of the antimicrobial, antiviral, immunomodulatory, immune-supporting or other effects was extracted from the papers and presented in a summary Table for each discussed plant-derived agent.

## 3. Results

The current study represents summarized information for the properties of *Echinacea* spp., *Pelargonium sidoides*, *Hedera helix*, *Thymus vulgaris*, *Althaea officinalis*, *Sambucus nigra*, *Zingiber officinale*, and *Curcuma longa* related to RTIs.

The clinical trials published on PubMed from 2000 to 2025 for both in vivo and in vitro studies of plants and plant-derived agents for URTIs and LRTIs are listed in Table 1.

The clinical trials included in this study involve plant-derived agents presented in various forms, including syrups, drops, teas, tablets, capsules, and essential oils. The targeted populations for these trials vary and include both adults and children. Additionally, the types of clinical trials conducted are diverse. These factors allow for a more logical organization of the findings rather than relying solely on statistical methods. Since this review aims to provide a broad conceptual overview rather than a point estimate of effect, formal statistical synthesis was not performed.

Our narrative review categorizes evidence by study type in four groups: (1)—Randomized trials, (2)—Non—randomized trials, (3)—Controlled trials, (4)—Uncontrolled trials. The randomized and placebo-controlled trials were 66 (70.2%). Six studies were non-randomized, and 22 were uncontrolled studies.

We assess the strength of the findings, utilizing Melnyk and Fineout-Overholt’s Levels of Evidence [34]. This hierarchical framework was used to classify the overall evidence-based quality of the clinical trials included in various sections. We employed it to organize and assess these trials into levels based on their design. The hierarchy identifies levels I through VII of evidence. In our study, all clinical trials fall into the following three levels:

- Level II: Well-designed Randomized Controlled Trials (RCTs).
- Level III: Controlled trials without randomization (Quasi-experimental).
- Level IV: Well-designed case–control or cohort studies.

Due to the diversity among the included studies, strictly categorizing them within a rigid hierarchy was not feasible. The Melnyk and Fineout-Overholt hierarchy was not intended to serve as an analytical tool for evaluating detailed quality aspects in this narrative review.

The described evidence levels refer to the degree of confidence in the reliability and consistency of research findings, based on the quality and design. A higher evidence level suggests that the current body of research strongly supports a particular conclusion or intervention.

**Table 1:** Clinical trials for *Echinacae* spp., *Pelargonium sidoides*, *Hedera helix*, *Thymus vulgaris*, *Althaea officinalis*, *Sambucus nigra*, *Zingiber officinale*, and *Curcuma longa* used in Respiratory Tract Infections (2000–2025).

Plant (Scientific Name)	Indications for Use	RTI Type ^1^ Most Common Use	Type of Study (Categorization) ^2^	Authors	Country	Evidence Level ^4^
*Echinacea* spp.	**Recurrent URTIs** (Sore throat Nasal congestion Cough) **Flu-like symptoms** (Runny nose Mild fever Body aches) **Immune support**	URTIs	Randomized, controlled and blinded CT ^3^ (1)	[35]	Switzerland	High to Moderate
			Randomized, double-blind, placebo-controlled (1)	[36]	Poland	
			Randomized, double-blind, placebo-controlled CT (1)	[37]	USA	
			Randomized, double-blind, placebo-controlled CT (1)	[38]	USA	
			Randomized, double-blind, placebo-controlled parallel group (1)	[39]	USA	
			Observational, prospective, monocentric CT (2)	[40]	Bulgaria	
			Randomized, controlled CT (1)	[41]	USA	
			Single-blind randomized CT (1)	[42]	Iran	
			Controlled, double blind, randomized CT (1)	[43]	Armenia	
			Randomized, double-blind, placebo-controlled, multicenter CT (1)	[44]	Israel	
			Comparative controlled study (3)	[45]	Russia	
			Parallel-group, randomized, double-blinded, placebo-controlled CT (1)	[46]	Armenia	
			Double-blind, randomized, placebo-controlled CT (1)	[47]	Indonesia	
			Comparative Study, in vitro (3)	[48]	Italy	
			Randomized, double-blind, placebo-controlled CT (1)	[49]	USA	
			Randomized, double-blind, placebo-controlled CT (1)	[50]	USA	
			Randomized, double-blind, placebo-controlled CT (1)	[51]	Korea	
			Randomized, double-blind, placebo-controlled trial (1)	[52]	Canada	
			Randomized, double-blind and placebo-controlled trial (1)	[53]	Canada	
			Randomized, double-blind, placebo-controlled, clinical dose–response CT (1)	[54]	Germany	
			Non-randomized, multicentre, nationwide, two-armed research study (2)	[55]	Germany	
*Pelargonium* *sidoides*	**URTI symptoms** (Runny nose Nasal congestion Sore throat) **Bronchitis symptoms** (Cough Difficulty clearing mucus Shortness of breath Thick bronchial secretions, Increased sputum)	URTIs/ LRTIs	Single-blind, randomized, placebo-controlled CT (1)	[56]	Turkey	High to Moderate
			Double-blind, placebo-controlled randomized CT (1)	[57]	UK	
			Randomized, double-blind, placebo-controlled, parallel-group (1)	[58]	Germany	
			Multicenter, randomized, double-blinded, active-controlled CT (1)	[59]	South Korea	
			In vitro experimental CT (3)	[60]	Switzerland	
			Randomized, double-blind, placebo-controlled CT (1)	[61]	Ukraine	
			Prospective, double-blind, parallel-group, placebo-controlled CT (1)	[62]	USA	
			Randomized, double-blind, placebo-controlled clinical dose-finding (1)	[63]	Germany	
			Comparative study, in vitro (3)	[64]	Italy	
			Comparative study, in vitro (3)	[65]	Lithuania	
			Multi-centre, prospective, open observational study (2)	[66]	Germany	
			Randomized, double-blind, placebo-controlled, dose-finding (1)	[67]	Germany	
			Multi-centre, randomized, double-blind CT (1)	[68]	Korea	
			Randomized, double blind, placebo-controlled CT (1)	[69]	Ukraine	
			Randomized, double-blind, placebo-controlled CT (1)	[70]	Germany	
			Randomized, double-blind, placebo-controlled CT (1)	[71]	Belgium	
			Randomized, double-blind, controlled CT (1)	[72]	Germany	
			Randomized, double-blind, placebo-controlled, multicentre CT (1)	[32]	Germany	
			Randomized, double-blind, placebo-controlled CT (1)	[73]	Russia	
			Randomized, double-blind, placebo-controlled CT (1)	[74]	Germany	
			Randomized double-blind, placebo-controlled CT (1)	[75]	Germany	
			Randomized, double-blind, placebo-controlled, multi-centre CT (1)	[76]	Germany	
			Randomized, Open-Label Study (1)	[77]	Germany	
			Prospective, open, multicentric, non-randomized CT (2)	[78]	Germany	
*Hedera* *helix* (Ivy leaf)	Productive cough, Persistent cough Irritative cough with mucus Acute bronchitis bronchial spasms	LRTIs	Randomized controlled CT (1)	[79]	Pakistan	Moderate
			Randomized, double-blind, placebo-controlled, multicenter CT (1)	[80]	Spain	
			Non-controlled, observational CT (4)	[81]	Germany	
			Open-label, prospective, randomized CT (1)	[82]	Serbia	
			Randomized, placebo-controlled, double-blind CT (1)	[83]	Germany	
			PMSS—Observational, non-interventional study (4)	[84]	Germany	
			Double-blind, randomized CT (1)	[85]	Germany	
			Randomized controlled CT (1)	[86]	Pakistan	
			Multicenter, randomized, double-blinded, active-controlled, parallel, therapeutic confirmatory CT (1)	[59]	South Korea	
			Multicenter, observational survey (2)	[87]	Poland	
			Multicenter, open-label, prospective, single-arm, observational study (4)	[88]	Korea	
			PMSS—Multicenter, non-interventional, observational (4)	[89]	Germany	
			Non-randomized, non-interventional, multicenter, open-label, post-authorization effectiveness study (PAES) (2)	[90]	Poland	
			Double blind, placebo-controlled, randomized cross-over study (1)	[91]	Germany	
			Uncontrolled, open, multicenter study (4)	[92]	Switzerland	
			Prospective, double-blind, placebo-controlled CT (1)	[93]	Germany	
			Prospective, open, multi-centre PMSS (4)	[94]	Uruguay	
			PMSS (4)	[95]	Germany	
*Thymus vulgaris* (Thyme)	Sore throat, Productive cough, Difficulty expectorating mucus, bronchial irritation, reduce bronchial spasms	URTIs/ LRTIs	Observational, prospective, uncontrolled study (4)	[33]	Germany	Moderate
			Randomized controlled CT (1)	[96]	Turkey	
			Uncontrolled, open, multicenter study (4)	[92]	Switzerland	
			Comparative in vitro analysis (3)	[97]	Germany	
			Prospective, double-blind, placebo-controlled multi-centre CT (1)	[98]	Germany	
*Althaea* *officinalis* (Marshmallow root)	Non-productive dry cough, Sore throat, Irritated pharynx/ larynx	URTIs	Randomized, open-label, multicenter, comparative study (1)	[99]	Ukraine	Low to moderate
			Uncontrolled, open, multicenter study (4)	[93]	Germany	
			Randomized controlled CT (1)	[86]	Pakistan	
			Randomized controlled CT (1)	[79]	Pakistan	
*Sambucus nigra* (Elderberry)	**URTI symptoms** Runny nose Nasal congestion Sore throat Sinus discomfort Cough **Immune support**	URTIs	Randomized, Double-Blind Placebo-Controlled CT (1)	[100]	Australia	High to Moderate
			Randomized, double-blind, placebo-controlled study (1)	[101]	Israel	
			Randomized, Double-Blind, Placebo-Controlled CT (1)	[102]	USA	
			Comparative in vitro study (3)	[103]	Germany/ Canada	
			Randomized Case–Control Study (1)	[104]	Italy	
			Comparative in vitro study (3)	[105]	Italy	
			Randomized CT (1)	[106]	Italy	
			Comparative in vitro study (3)	[107]	USA	
			Comparative in vitro study (3)	[108]	Israel	
*Zingiber officinale* (Ginger)	**URTI symptoms** Sore Throat, Cough, Inflammation **Immune support**	URTIs	Randomized double-blind placebo-controlled CT (1)	[109]	Iran	High to Moderate
			Randomized placebo-controlled CT (1)	[110]	Iran	
			Randomized, placebo-Controlled, double-Blind Crossover CT (1)	[111]	Turkey	
			Randomized, placebo-Controlled CT (1)	[112]	Iran	
			Single centre, randomized, double-blind, placebo-controlled CT (1)	[113]	Iran	
			Comparative in vitro study (3)	[114]	South Korea	
			Single centre, randomized, 2-arm, parallel group, double blind, controlled CT (1)	[115]	India	
			Randomized, double-blind, placebo-controlled CT (1)	[116]	UK	
*Curcuma longa* (Turmeric)	**RTIs symptoms** Throat irritation Bronchial irritation **Immune support** Immune modulation	URTIs/LRTIs	Randomized, double-blind CT (1)	[117]	Iran	High
			Randomized, double-blind CT (1)	[118]	Iran	
			Randomized controlled CT (1)	[119]	China	
			Randomized, Double-Blind, Placebo-Controlled CT (1)	[120]	Japan	
			Randomized Triple-blind CT (1)	[121]	Iran	

^1^ RTI—respiratory tract infection; ^2^ Type of study (categorization): (1)—Randomized CT (2)—Non-randomized CT (3)—Controlled CT (4)—Uncontrolled CT; ^4^ Evidence level: High (very confident level); High to Moderate (consistent evidence from RCT); Moderate (mixed RCT results); Low to moderate (limited or inconsistent evidence); ^3^ CT—Clinical trial.

## 4. Plant-Derived Agents

### 4.1. Echinacea spp.

**Figure 2:**
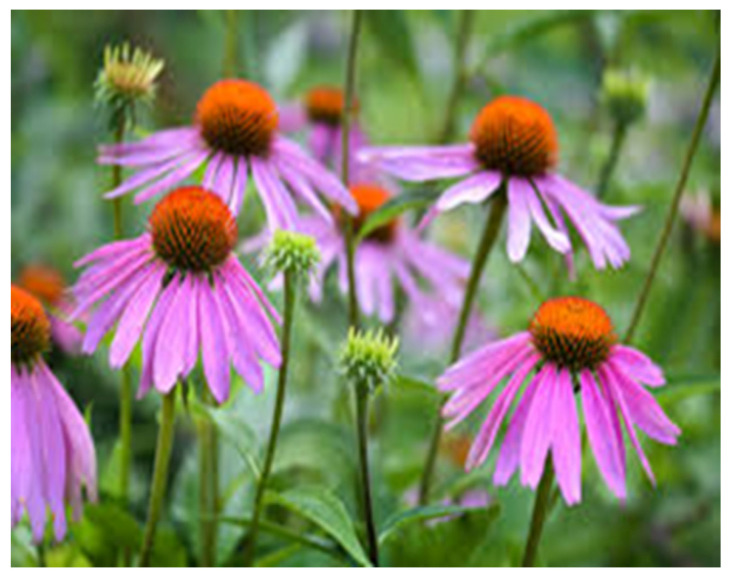
*Echinacea* spp.

*Echinacea* (Figure 2) is a genus that encompasses nine species: *E. angustifolia*, *E. atrorubens*, *E. laevigata*, *E. pallida*, *E. paradoxa*, *E. purpurea*, *E. sanguinea*, *E. simulata*, and *E. tennesseensis*, belonging to the *Asteraceae* family. Of particular interest are *Echinacea purpurea*, *E. angustifolia*, and *E. pallida*, which have been shown to possess various biological properties that may support the body’s defence mechanisms during RTIs and may help to reduce the duration of symptoms. These benefits are primarily attributed to their bioactive compounds, including alkamides, caffeic acid derivatives, polysaccharides, and glycoproteins [122,123].

The dosages and methods of extraction for *Echinacea* varied significantly across the studies included. The research summarized in Table 1 utilized different parts of the herb, including the root, the whole plant, and the aerial parts, and employed various preparation methods. *Echinacea* was administered in several forms, such as pressed juice, hydroalcohol extracts, dry herb capsules, and infusions [123].

A formulation containing alkamides, cichoric acid, and polysaccharides, prepared from freshly harvested *Echinacea purpurea* plants, was used in a randomized, double-blind, placebo-controlled trial. 282 subjects aged 18–65 years with a history of two or more colds in the previous year were recruited for this study. The subjects were randomized to receive either echinacea or a placebo. They were instructed to start *Echinacea* or a placebo at the onset of the first symptom related to a cold and were examined on days 3 and 8 of their cold. The results revealed that a total of 128 subjects contracted a common cold, and the total daily symptom scores were found to be around 23% lower in the echinacea group than in the placebo group. The response rate to treatments was greater in the echinacea group. Early intervention with a standardized formulation of echinacea resulted in reduced symptom severity in subjects with naturally acquired upper respiratory tract infection [52].

Herbal preparations made from the leaves and roots of *E. purpurea* have been shown to increase the number of phagocytic cells in the spleen and bone marrow, acting as phytoimmune modulators or enhancers of the immune system [124]. In vitro studies provide support for claims of immune modulation, indicating that this effect may be related to changes in the activity of polymorphonuclear neutrophil granulocytes, macrophages, and cytokine production, including IL-1, IL-6, and TNF-α [125,126]. Some studies suggest an enhancement of natural killer (NK) cell activity. It modulates inflammatory pathways, potentially preventing excessive immune responses. This dual immune-stimulating and immune-regulating action may help the host respond more effectively to viral respiratory infections [127,128].

The clinical trials conducted on *Echinacea* spp. can be categorized as follows: 19 of these studies were randomized controlled clinical trials (CTs), demonstrating strong scientific rigour, while three were non-randomized, which still provide important insights. The evidence level regarding the effectiveness of *Echinacea* spp. is classified as high to moderate evidence, supported mainly by randomized and controlled CTs. Its benefits seem to be more consistent in treating viral URTIs. In vitro studies and other clinical trials suggest that *Echinacea* is active against various viruses, including Rhinoviruses, Influenza viruses, Parainfluenza viruses, and Respiratory Syncytial Virus (RSV), rather than against bacterial infections [129,130,131].

The primary benefit of RTIs is indirect, through immune modulation rather than direct pathogen eradication. Clinical studies indicate that *Echinacea* may reduce the severity of symptoms, particularly symptoms associated with inflammation, such as throat irritation and may alleviate the nasal congestion and cough (Table 2). Some trials have shown that using *Echinacea* prophylactically may decrease the frequency of recurrent URTIs, particularly in children who are prone to repeated infections [35,36,132].

This effect is thought to be related to enhanced mucosal immunity. *Echinacea* may influence upper airway mucosal immunity by supporting the local immune response and potentially increasing secretory IgA levels [39]. A randomized CT evaluated the impact of *E. purpurea* on mucosal immunity and URTI. Thirty-two participants with URTI provided saliva samples and took either a placebo or *Echinacea* for four weeks. The s-IgA and saliva flow were measured before and after the test. The results after four weeks demonstrated that only the control group showed a decrease in s-IgA. These findings indicate that *Echinacea* may help maintain mucosal immunity and shorten RTI duration [39]. This property may be relevant for the prevention and early-stage management of URTIs.

### 4.2. Pelargonium sidoides

**Figure 3:**
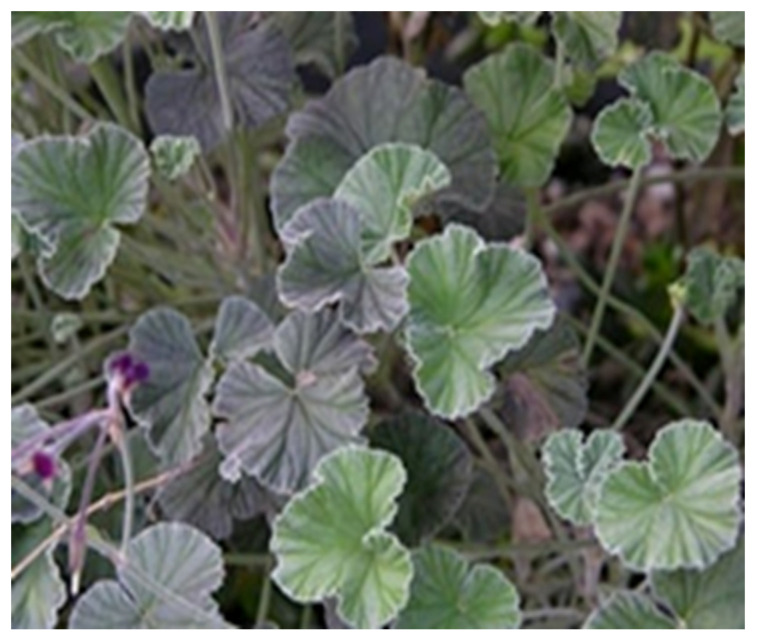
*Pelargonium sidoides*.

*Pelargonium sidoides* (Figure 3) is a perennial plant that belongs to the *Pelargonium* genus within the *Geraniaceae* family. It is primarily found in Southern Africa [136]. The pharmacological research began with a standartized extract of *P. sidoides* known as EPs^®^ 7630 (Umckaloabo^®^) in the late 20th century [137].

EPs^®^ 7630 is an extract from the roots of *P. sidoides* with a drug ratio 1:8–10 using 11% ethanol as an extraction solvent [138,139]. Its pharmacological activity is attributed to bioactive compounds such as coumarins, phenolic acids, flavonoids, and proanthocyanidins. *P. sidoides* composition comprises metabolites, such as carbohydrates, amino acids, peptides, and minerals, which represent about 30% of the composition of EPs^®^ 7630 [139]. Highly oxygenated coumarins and proanthocyanidins correspond to approximately 40% of the extract’s content, and it is believed to be the main components responsible for the extract’s effects [139].

A trial with 199 adults diagnosed with COPD stages II/III and receiving standard treatment according to the Global Initiative for Chronic Obstructive Lung Disease (GOLD) revealed results after being randomly assigned to add-on therapy with EPs^®^ 7630 or placebo for 24 weeks. More pronounced improvement under EPs 7630 than under placebo was noted in the study, including symptom severity score of cough, sputum production, and sternal pain while coughing. The study stated that add-on therapy with EPs 7630 led to an improvement in adult patients with COPD compared to placebo, while showing a good long-term tolerability [58].

The specific metabolites in Pelargonium extract interact with bacteria, effectively demonstrating either bacteriostatic or bactericidal effects. Several Gram-positive bacterial species have been evaluated, such as *Staphylococcus aureus* [65,140], *Streptococcus pneumoniae* [141,142,143], *Enterococcus faecalis* [64], *Streptococcus pyogenes* [64], *Mycobacterium tuberculosis* [144], and *Staphylococcus epidermidis* [145]. Gram-negative bacteria *Escherichia coli* [146,147], *Klebsiella pneumoniae* [148], *Pseudomonas aeruginosa* [149], and *Porphyromonas gingivalis* [150] were also studied.

*P. sidoides* exhibits antifungal properties by inhibiting laccase enzyme, inducing fungal cell death through structural disruption, and reducing capsule size [64,151]. In terms of antiviral activity, the extract may affect influenza viruses, respiratory syncytial virus (RSV), and rhinoviruses (Table 3). It causes viral death and inhibits surface glycoproteins like hemagglutinin and neuraminidase, thereby preventing viral attachment and replication [152,153].

The anti-adhesive and mucokinetic effects of *P. sidoides* are particularly relevant in lower RTIs characterized by mucus clearance. It prevents bacterial adhesion to human cells by interacting with bacterial adhesins and human cell membrane glycoproteins [64]. Moreover, the extract is known for its immunomodulatory effects, primarily through gene expression modulation, which leads to increased cytokine expression, including IL-1, IL-6, IL-10, IL-2, TNF-α, and IFN. These changes enhance phagocytosis and intracellular killing [154].

The anti-inflammatory activity contributes to symptomatic relief, including reduced cough, and demonstrated efficacy in duration with acute bronchitis and other lower respiratory tract infections [155].

The clinical trials in Table 1 for *P. sidoides* were categorized as follows: 22 CTs were randomized controlled trials, while two CTs were non-randomized, but offered valuable additional insights. They demonstrated high to moderate evidence level, according to the Melnyk and Fineout-Overholt’s Levels of Evidence scheme.

Conclusions about effectiveness cannot always be strictly categorized. A number of factors influence the patient’s condition, as well as the different forms in which herbal preparations are taken and the duration of intake.

In a double-blind, placebo-controlled trial with a *Pelargonium*-derived agent, 103 patients with cough lasting 21 days or less due to acute bronchitis were recruited from UK general practices and completed a diary. Practices were cluster-randomized to liquid or tablet preparations, and patients were individually randomized to receive either a plant-derived agent or a placebo. The results showed that 41% of patients took antibiotics. The breakdown was: Pelargonium agent in liquid form 48%, placebo liquid 23%, Pelargonium agent in tablet form 48%, and placebo tablet 50%. Most patients followed the medication schedule, taking a median of 19 out of 21 doses in the first week, with an interquartile range of 18 to 21 across all groups. This RCT with a Pelargonium-derived agent for the treatment of acute bronchitis demonstrated low data attrition [57].

Other clinical trials indicate that variations in the concentration of active compounds in the same plant can lead to significantly different biological effects. One of the included studies in Table 1 was a dose-finding trial, carried out to evaluate the therapeutic benefits of EPs-7630 in children and adolescents with RTIs. In the study, 400 patients aged 6 to 18 years were randomized to receive daily doses of either 30 mg, 60 mg, or 90 mg of EPs-7630 or a placebo. The main focus of the study was to assess changes in the Bronchitis Severity Score (BSS) from day 0 to day 7. After one week of treatment, the groups receiving 60 mg and 90 mg of EPs-7630 demonstrated significant improvements in their BSSs compared to the placebo group. Key symptoms, including coughing, sputum production, and rales during auscultation, showed notable enhancement under the treatment. This study highlights EPs-7630 as a promising option for managing acute bronchitis in 6 to 18-year-olds, revealing faster onset of relief, a shorter duration of bed rest, and higher overall satisfaction with the treatment outcomes [63].

### 4.3. Hedera helix

**Figure 4:**
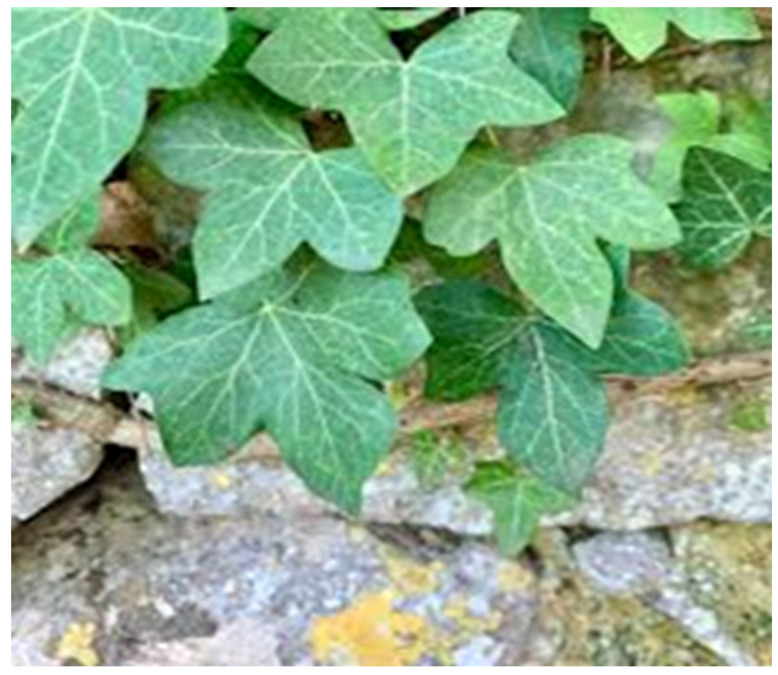
*Hedera helix*.

*Hedera helix* (Figure 4) is a part of the *Araliaceae* family. The common name is ivy leaf, and it contributes to antitussive effects, decreased cough intensity and effects in chronic lung diseases. *Hedera helix* is also linked in different studies to activation of immunoregulatory mechanisms and helps in the prevention of excessive immune activation, limiting inflammation and tissue damage. It is frequently used in pediatric cough syrups, has expectorant and bronchodilatory properties and may help reduce cough severity in bronchitis and other LRTIs [87,156,157].

Numerous clinical studies [83,158,159,160] have demonstrated the efficacy of *H. helix* in alleviating symptoms of acute and chronic RTIs by reducing coughing, bronchospasms, and secretion. Ivy leaf extract contains saponins, flavonoids, phenolic acids and polyacetylenes, which are believed to have expectorant properties [161].

In vitro studies indicate that the saponin, α-hederin, is one of the main active compounds in ivy leaf extracts. α-hederin is thought to inhibit the internalization of β2-adrenergic receptors on alveolar type II cells and human airway smooth muscle cells, resulting in increased β-adrenergic responsiveness in the respiratory tract [162,163]. Ivy leaf dry extract EA 575 is the first phytomedicine for which biased β2-adrenergic receptor activation has been demonstrated [164]. Other in vitro studies have demonstrated that G protein-biased signalling pathways lead to a significant elevation in cyclic adenosine monophosphate (cAMP) levels within cells. This increase in cAMP triggers the secretion of pulmonary surfactant, a substance that plays a critical role in reducing surface tension in the alveoli of the lungs. The rise in cAMP levels contributes to a reduction in the viscosity of mucus, facilitating improved mucociliary clearance and bronchodilation. As a result, airway resistance decreases, clinically presenting as secretolytic and bronchospasmolytic effects [160,163,165]. Furthermore, in vitro studies indicate that EA575 may have anti-inflammatory properties [166,167].

Both saponins and polyphenols possess anti-inflammatory properties through mechanisms like the direct inhibition of pro-inflammatory cytokine production and modulation of arachidonic acid metabolism. Polyphenols also stimulate natural killer (NK) cells and promote anti-inflammatory cytokines (Table 4) [168,169,170].

A study [157] demonstrates for the first time that EA 575^®^ is a potent immunomodulator. The extract modulates T-cell immune responses through complex mechanisms involving dendritic cells. Matured dendritic cells treated with EA 575^®^ decrease the production of immunogenic cytokines (e.g., IL-12 family) and reduce T-cell proliferation. Additionally, they inhibit Th1, Th17, Th9, Th21, and pro-inflammatory immune responses. These immunomodulatory effects are linked to the induction of various subsets of tolerogenic and exhausted Th cells. Lower concentrations of EA 575^®^ are more immunomodulatory, while higher concentrations are more anti-inflammatory.

Another study [171] highlights the impact of EA 575^®^ on A2B adenosine receptor signalling and the subsequent release of IL-6, by inhibiting A2BAR signalling, which plays a crucial role in chronic inflammatory pulmonary diseases [172]. A2BAR signalling acts on cellular pathways activated by the adenosine A2B receptor when it binds adenosine, released during inflammation or low oxygen conditions. The results may help explain the beneficial effects of EA 575^®^ in treating bronchospasm and enhancing bronchodilation when used alongside α-Hederin. It increases the availability of β_2_-adrenergic receptors, which enhances bronchodilation, potentially reducing bronchial spasms and improving airflow.

The clinical trials for *H. helix* were categorized into three groups: nine randomized CTs, two non-randomized trials, and seven uncontrolled trials. Overall, the evidence from these trials was not strong enough to be considered definitive. We observed mixed results from both the randomized and non-controlled observational studies (Table 1).

There are clinical trials investigating various forms of ivy plant extracts. One double-blind, randomized study evaluated the efficacy and tolerability of a soft extract made from ivy leaves, using a solvent of 50% (*v*/*v*) ethanol and propylene glycol (98:2). The ethanol in this fluid extract was subsequently removed through vacuum distillation, and its effects were compared to those of a dry ivy leaf extract. The study involved 590 patients diagnosed with acute bronchitis, who were assigned to receive either the test product or the comparator for a duration of 7 days. The Bronchitis Severity Score (BSS) showed a gradual decrease in both treatment groups from Day 1 to Day 7. Participants started with mean BSS values between 6.2 and 6.3 (±1.2) and finished the study with mean BSS values ranging from 1.4 to 1.6. Significant improvements were observed in symptoms such as cough, sputum production, wheezing, chest pain during coughing, and dyspnea in both groups. The soft extract of ivy leaves proved to be as effective as the comparator extract in alleviating symptoms of acute bronchitis [85]. Other studies investigate the combination of *H. helix* with other plant-derived agents to evaluate the effectiveness and tolerability of a fixed combination of fluid extracts from thyme and ivy leaves, compared to a matched placebo, in patients suffering from acute bronchitis with a productive cough [92,93]. In one double-blind, placebo-controlled, multicenter study, 361 outpatients with acute bronchitis and a Bronchitis Severity Score (BSS) of 5 or higher were randomly assigned to receive either the thyme-ivy combination syrup or a placebo syrup for 11 days. Results showed that participants in the thyme-ivy combination group experienced a 50% reduction in coughing fits two days earlier than those in the placebo group. While symptoms of acute bronchitis (as measured by the BSS) improved rapidly in both groups, the resolution of symptoms was significantly faster in the group receiving the thyme-ivy combination treatment compared to the placebo group [93].

**Table 4:** Properties of *Hedera helix* relevant to Respiratory Tract Infections.

Plant	Properties	Contribution	References
*Hedera helix*	Immunomodulatory properties	Stimulates NK cells, promotes anti-inflammatory cytokines, modulates T-cell immune response	[157,168,169,170]
	Antibacterial properties	Minor activity	[173]
	Antiviral activity	Enhanced activity	[174,175]
	Anti-inflammatory effects	Reduces pro-inflammatory mediator release Limits the irritation of the respiratory mucosa	[166,167]
	Expectorant activity by mucus clearance	Reduces mucus viscosity Enhances mucociliary transport Facilitates expectoration	[160,163,165]

### 4.4. Thymus vulgaris

**Figure 5:**
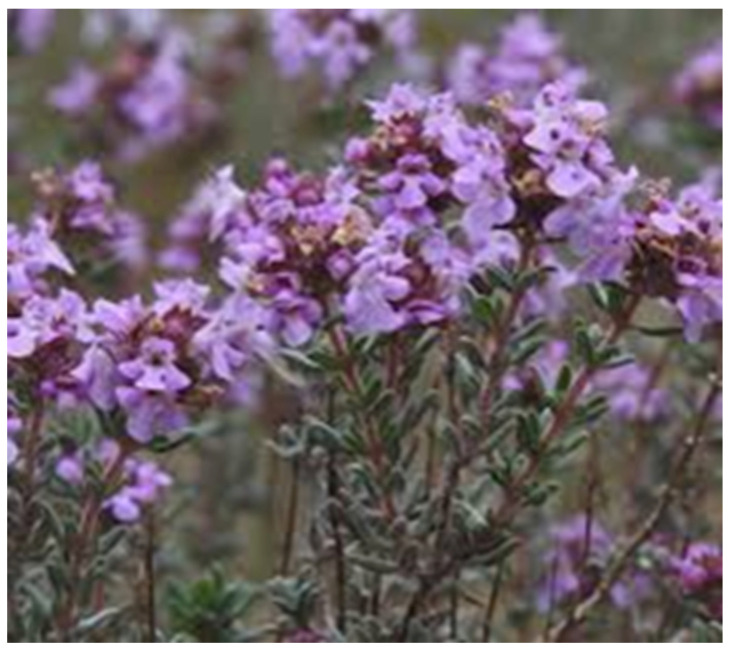
*Thymus vulgaris*.

The common name of *Thymus vulgaris* is thyme (Figure 5). It is a perennial, woody-based subshrub in the genus *Thymus,* belonging to the *Lamiaceae* family. Thyme exhibits expectorant, antitussive, bronchospasmolytic, and antimicrobial properties that contribute to its established role in the symptomatic management of RTIs, particularly acute bronchitis [31,176].

Thyme has a strong composition of vitamin C, beta-carotene, and vitamin A [177]. Several other vitamins are also found in thyme, including vitamin B6, vitamin E, folic acid, and vitamin K [178]. Phenolic acids and flavonoid antioxidant compounds such as luteolin, quercetin, apigenin, ferulic acid, zeaxanthin, naringenin, and thymonin are verified as flavonoid compounds [177]. Chemical constituents of thyme, like thymol, carvacrol, eugenol, linalool, apigenin, rosmarinic acid, and *p*-cymene, have their specific biological action [179]. Several activities, confirmed and exhibited by apigenin, are antiviral, anti-inflammatory, anticarcinogenic, antimutagenic, and antioxidant. Monoterpene thymol is a crystalline phenolic compound, considered the major constituent of thyme essential oil, which also represents strong antioxidant, antiseptic, antifungal, and antibacterial properties [180]. Carvacrol is an important monoterpene compound with several effects, reported as acetyl cholinesterase inhibitory action, antimicrobial, anti-inflammatory, and antithrombotic [181].

The immunomodulatory effect of thymol is associated with an enhancement of phagocytosis, which plays an essential role in the first line of immunity. Thymol increases membrane fluidity, thereby boosting the activity of macrophages (Table 5) [182].

In vitro studies demonstrated that carvacrol helps in the expression and production of pro-inflammatory intermediates. It has been revealed that thymol upturns superoxide anion production by improving respiratory burst, through reducing the release of pro-inflammatory cytokines IL-1β, IL-6 and TNF-α in lipopolysaccharide-stimulated cells [183]. Methanolic extract of thyme was classified as a potent agent in inhibiting nitric oxide release from lipopolysaccharide -activated macrophages [184]. Furthermore, studies reported thyme is capable of regulating the anti-inflammatory and pro-inflammatory cytokines along with other signalling pathways in tissues [185], as well as immune cell function improvement [186].

The inhibitory action of thymol was investigated in Gram-negative and Gram-positive bacteria [187]. The antibacterial effect involves the disruption of microbial cell membranes, which increases permeability and then leads to cell lysis. Thymol affects microbial gene expression and metabolic processes, in addition to cell membranes. Studies have reported that thymol exposure can amend the expression of genes linked with stress-related reactions. The alteration in gene expression can make infections less able to survive ecological stressors, which would increase the efficacy of thymol. Thymol targets various microbial physiology elements, instead of depending on traditional antibiotic processes, which makes it a practical technique for treating antimicrobial resistance [188].

Antitussive and spasmolytic action has been attributed to carvacrol and thymol [189]. Through the inhibition of histamine receptors and acetylcholine, flavonoids in thyme appear to relax ileal and tracheal smooth muscles in animal models. In vitro utilization of the extract and volatile oil of thyme applies soothing and relaxing effects on ileal as well as tracheal smooth muscles through the inhibition of contractions, which could also be subjected to the presence of flavone aglycones [190].

Thymol and carvacrol have a considerable effect on the lungs, which provokes the secretion of the mucous membrane and increases ciliary movement in bronchial epithelia [191]. The effectiveness and tolerability of a combination of dry extracts from thyme herb and primrose root were assessed in adults with acute bronchitis featuring a productive cough. This evaluation took place in a prospective, double-blind, placebo-controlled multicenter clinical trial. The study recruited 361 outpatients randomly assigned to an 11-day treatment regimen (one tablet taken three times daily) with either the thyme-primrose combination (n = 183) or a placebo (n = 178). Results showed a significant reduction in coughing for the group receiving the thyme-primrose combination compared to the placebo group. They achieved a 50% reduction in coughing approximately two days earlier than those in the placebo group. While symptoms of acute bronchitis (as measured by the BSS) improved quickly in both groups, the improvement was more rapid in the thyme-primrose combination group [98].

Thymol’s antibacterial and anti-inflammatory properties have been studied in clinical trials regarding respiratory tract-related diseases, particularly concerning chronic obstructive pulmonary disease (COPD). Studies about thymol suggest that it may help improve lung function as well as minimize inflammation of airways [192,193].

A randomized controlled trial involving 140 COVID-19 patients (70 in the experimental group and 70 in the control group) found that thyme oil aromatherapy is effective in reducing symptoms and improving hemodynamic parameters in these patients [96]. The experimental group inhaled thyme oil three times a day for five days, while the control group received only routine treatment. At the end of the five-day period, symptoms and hemodynamic parameters were measured for both groups. The findings indicated that thyme oil significantly reduced body temperature, pulse rate, and respiratory rate. It also had a positive effect on the regulation of pH levels, decreasing CO_2_ and increasing O_2_ significantly. Although there was some improvement in symptoms like nausea, vomiting, runny nose, and loss of taste or smell, these effects were not statistically significant. This trial recommended thyme oil as a non-pharmacological treatment option for COVID-19 patients [96].

Overall, in our review, we identified a small number of CTs involving *Thymus vulgaris* compared to other plant-derived agents. These trials included three randomized CTs and two non-randomized controlled trials, providing a moderate level of evidence.

**Table 5:** Properties of *Thymus vulgaris* relevant to Respiratory Tract Infections.

Plant	Properties	Contribution	References
*Thymus* spp.	Immunomodulatory properties	Promotes phagocytosis and anti-inflammatory cytokines	[182,183]
	Antibacterial properties	Disruption of microbial cell membranes, cell lysis. Affects microbial gene expression and metabolic processes	[188]
	Antiviral activity	Inhibits the growth of certain respiratory viruses in vitro	[176,194]
	Anti-inflammatory effects	Reduces pro-inflammatory mediators. Antioxidant protection to the respiratory epithelium.	[180,183]
	Antitussive and spasmolytic action	Thymol and carvacrol reduce the mucus viscosity, enhance bronchial secretions, promote expectoration	[189,191]
	Broncho-spasmolytic activity	Smooth muscle relaxant effects Reduces bronchial spasm Improves airflow	[190]

### 4.5. Althaea officinalis

**Figure 6:**
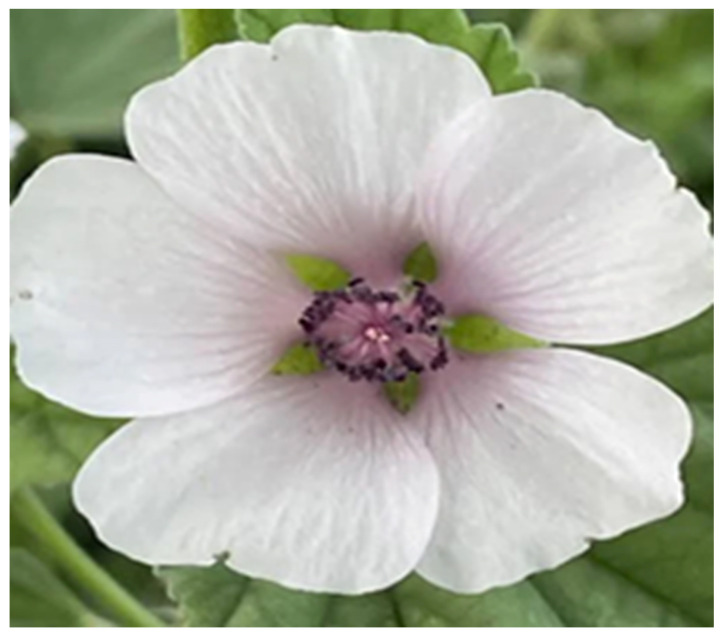
*Althaea officinalis*.

*Althaea officinalis* (Figure 6) from the *Malvaceae* family, also called marshmallow, is known as a medicinal plant for the treatment of the irritation of laryngopharyngeal mucosa, pharyngitis, tracheobronchitis, excruciating cough, and shortness of breath. Phytohustil^®^ is one of the most common herbal medicinal products containing root extract of *A. officinalis*, commonly used for the treatment of mucous membrane irritations in the mouth and throat and the dry cough associated with this [195,196].

Many compounds have been extracted from *A. officinalis*, including starch, pectins, saccharose, mucilage, flavonoids, caffeic acid, *p*-coumaric acid, isoquercitrin, coumarins, phytosterols, tannins, as well as many amino acids [197,198]. The root extract of *A. officinalis* contains water-miscible polysaccharides (acidic polysaccharides), mostly galacturorhamnans, arabinans, glucans, and arabinogalactans [199]. Rich in mucilage, marshmallow root forms a protective layer over irritated mucosa, helping to relieve dry cough and sore throat in URTIs [195].

The common oral use of marshmallow root against dry cough caused by pharyngeal and mucosal irritation is related to the bio-adhesive properties of the polysaccharides to the epithelial mucosa, which protects the cells from mechanical irritations and microbial invasion [200].

A randomized clinical trial revealed a promising intervention in children with cough and cold symptoms. The CT involved 220 patients, with 110 participants receiving a syrup made from marshmallow and mustard seeds, combined with ivy leaf extracts. The other 110 participants received a placebo. The findings revealed that the new combined treatment was not only effective but also demonstrated a high level of safety and tolerability among the diverse patient population, which included children as young as 3 years old up to adolescents over the age of 15 [86]. Another randomized controlled trial shows similar results in the same age group, demonstrating the effectiveness of a marshmallow and ivy leaf combination delivered in granule form [79].

In vitro investigations showed a significant anti-oxidant and anti-inflammatory activity of REAo in MΦ, with additional effects on cellular integrity and migratory capacity [201,202]. Migration of polymorphonuclear leukocytes (PMNs) is expected and followed by macrophage accumulation in response to tissue injury or infection, and characterized by local production of cytokines [203].

Bioactive low-molecular-weight compounds in marshmallow, as flavonoid-O-sulfoglycosides, are involved in the formation and regulation of the extracellular matrix in the mucosal tissue [200]. This connection can trigger cell–matrix interactions and subsequent migration, cytokine signalling, as well as leukocyte activation in both normal and pathological conditions [202].

After migration, tissue-resident macrophages ingest bacteria, dead cells and recognize lipopolysaccharides (LPS), which stimulates the synthesis and secretion of pro-inflammatory cytokines, such as TNF-α, IL6, IL-1β, etc. Secretion of cytokines is an important component of host defence, allowing the immune system to detect and respond to small quantities of LPS in the early stages of bacterial infection (Table 6) [204,205,206].

The marshmallow root also exhibited strong antioxidant activity, as well as effective reducing power, free radical/superoxide anion radical scavenging, and metal chelating activities [201]. Studies suggest that such extracts may be involved in the resolution of inflammation via anti-oxidative activity and phagocytosis regulation [201].

We found a small but significant number of clinical trials focused on *A. officinalis*. Among these, three were randomized controlled trials, and one was an uncontrolled study, categorized as moderate evidence.

**Table 6:** Properties of *Althaea officinalis* relevant to Respiratory Tract Infections.

Plant	Properties	Contribution	References
*Althaea officinalis*	Immunomodulatory Properties	Promotes anti-inflammatory cytokines and macrophage accumulation	[203]
	Antibacterial properties	Activity in the early stages of bacterial infection	[204,205]
	Antiviral activity	Minor activity	[196]
	Anti-inflammatory effects	Reduces pro-inflammatory mediator releasing Limits irritation of respiratory mucosa	[200,201,202]
	Expectorant activity by mucus clearance	Reduces mucus viscosity Facilitates expectoration	[195]

### 4.6. Sambucus nigra

**Figure 7:**
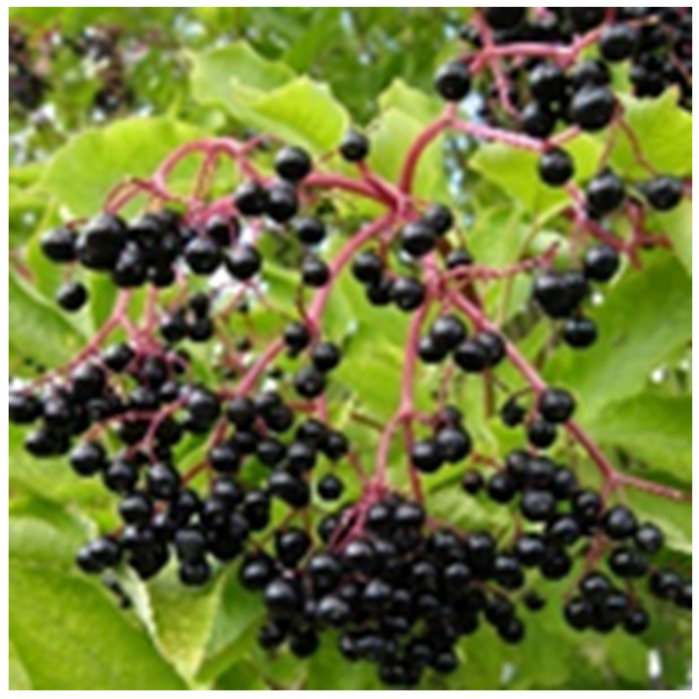
*Sambucus nigra*.

*Sambucus nigra*, is a species complex of flowering plants in the family *Viburnaceae*, commonly known as Elder (Figure 7). Elderberry extracts primarily exhibit antiviral and immunomodulatory effects and are commonly used to reduce symptom duration in viral URTIs [207,208].

The berries of *S. nigra* are dark violet-black, growing in clusters and owe their colour to anthocyanins, a group of phenolic compounds that are considered the active constituents of the fruits among flavonoids [209,210]. Elderberries contain an abundance of phenolic compounds, including *p*-hydroxybenzoic acid, protocatechuic acid, quinic acid, and chlorogenic acid; the anthocyanin, cyanidin-3-O-β-D-glucoside; other flavonoids, such as quercetin, quercetin-3-O-β-D-glucoside, rutin, and tannins [211,212,213]. *S. nigra* berries also contain α-linolenic acid, linoleic acid [214], mucilage and hydroxycinnamic acid derivatives. Elderberries also contain a variety of vitamins (A, B1, B2, B6, B9, C and E), elements Cu, Zn, Fe, minerals K, Ca and Mg, and phytochemicals such as carotenoids, phytosterols and polyphenols.

Different studies using a liquid elderberry extract reported antibacterial [215] and antiviral activities in vitro [216], and a beneficial effect on the severity and duration of cold and flu-like symptoms [100,217,218]. Elderberry exhibits mostly notable antiviral activity against respiratory viruses. It inhibits viral attachment and entry into host cells and reduces viral replication in vitro. Clinical trials demonstrated activity against *Influenza A* and *B* viruses and other respiratory viruses. These effects are most relevant during the early phase of viral URTIs [219,220].

A study examined the effectiveness and safety of oral elderberry extract for treating *Influenza* A and B virus infections. It represents randomized, double-blind, placebo-controlled trial during the influenza season with sixty patients, aged 18 to 54 years, who had been experiencing influenza-like symptoms for 48 h or less. The participants received either 15 mL of elderberry syrup or placebo syrup four times a day for five days. The results indicated that symptoms were relieved, on average, four days earlier in the group receiving elderberry extract compared to those receiving the placebo. The use of rescue medication was significantly lower among patients taking elderberry extract. The trial recommended elderberry extract as an effective, safe, and cost-effective treatment for influenza [101].

Elderberry has recently become popular due to its potential antioxidant [221], anti-inflammatory [222], immune-modulating, as well as antidepressant [223] and antidiabetic properties [224].

The polyphenols of the elderberry are involved in the anti-inflammatory effects and may reduce the inflammatory mediator release and alleviate nasal congestion, sore throat, and systemic symptoms. Anthocyanins provide strong antioxidant activity, which may protect respiratory epithelial cells from oxidative damage and support mucosal barrier integrity during infection (Table 7). In traditional medicine, elderberry has been used to reduce fever and relieve headache, fatigue, and myalgia associated with viral RTIs [220,225,226].

A real-world study during the COVID-19 pandemic era disclosed results in children with recurrent respiratory infections with a supplement containing *S. nigra* extract, β-glucan, Zinc, and Vitamin D3. Two hundred and ninety-eight children with RRI were enrolled in this study. The food supplement was randomly prescribed to 160 children with RRI daily for 4 months (active group), and a control group of 138 children with recurrent RI treated only with standard therapy for RI. The trial indicated that the tested supplement containing *S. nigra* might safely prevent RI episodes, is well-tolerated and reduces RI duration. The children in the active group experienced shorter RI duration during the treatment and follow-up phases [106].

Additionally, *S. nigra* modulates immune responses by enhancing cytokine production involved in antiviral defence and supporting in the activation of immune cells involved in pathogen clearance [108].

The clinical trials in Table 1 of *S. nigra* related to respiratory tract infections demonstrated a high to moderate level of evidence. All identified CTs were randomized, double-blind, placebo-controlled trials, along with a few comparative in vitro studies.

### 4.7. Zingiber officinale

**Figure 8:**
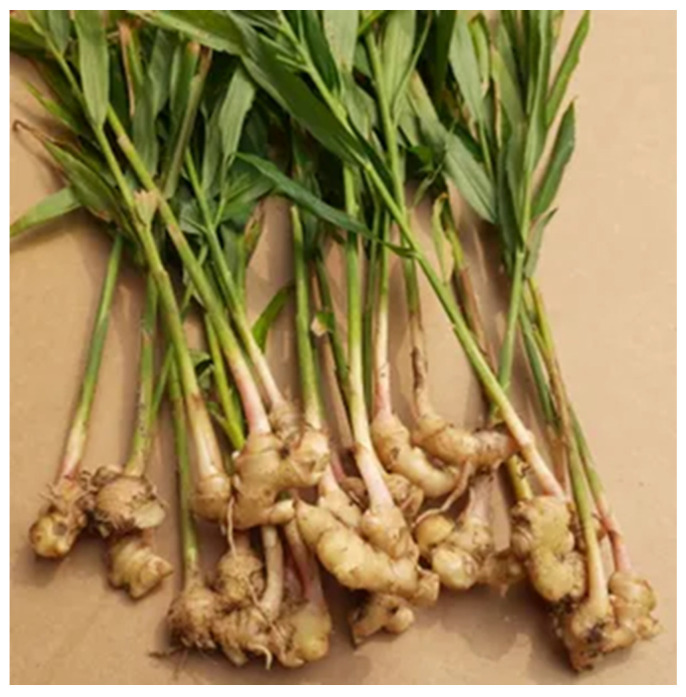
*Zingiber officinale*.

*Zingiber officinale,* or Ginger, is a flowering plant that belongs to the *Zingiberaceae* family and the *Zingiber* genus (Figure 8). Renowned for its rhizome, or underground stem, ginger has been utilized not only as a flavorful spice but also as a potent herbal remedy [228]. Ginger root is used to attenuate and treat several common diseases, such as respiratory [229], neurodegenerative diseases [230], cardiovascular diseases [231], obesity [232], diabetes mellitus [233], and chemotherapy-induced nausea and emesis [234]. In relation to respiratory diseases, it can significantly impact headaches, colds, nausea, and vomiting. Ginger is rich in bioactive components, particularly phenolic and terpene compounds [235]. The phenolic compounds in ginger are mainly gingerols, shogaols, and paradols. In fresh ginger, 6-, 8-, and 10-gingerols are the major polyphenols. When ginger undergoes heat treatment or extended storage, gingerols can be converted into shogaols. Through hydrogenation, shogaols can be transformed into paradols [236].

Different research indicates that ginger tea or extracts can alleviate symptoms of URTIs by reducing throat irritation, coughing, nasal congestion and sneezing [237]. Fresh ginger has been found to have antiviral effects against the human respiratory syncytial virus (RSV). Using ginger along with honey or lemon has been shown to shorten the duration of common cold symptoms in clinical settings [238,239]. Clinical observations indicate that ginger may assist in alleviating post-viral respiratory inflammation, including post-COVID symptoms, and can help relieve persistent cough, inflammation, and breathing difficulties [109,240].

In general, ginger and its active compounds have been found to be effective in alleviating inflammation. Ginger has the ability to reduce swelling and inflammation in the bronchial passages and can stimulate expectoration in the bronchial lining. It does this by inhibiting proteins and enzymes such as cyclooxygenase and lipoxygenase, which can help the body produce and clear mucus more effectively [241,242,243].

A series of studies showed that ginger and its active constituents possessed anti-inflammatory activity mainly related to phosphatidylinositol-3-kinase, protein kinase B, and the nuclear factor kappa -enhancer of activated B cells (NF-κB). Among the shogaols, 6-shogaol showed protective effects against TNF-α in human cell models. In addition, 6-dehydroshogaol was revealed to be more potent than 6-shogaol and 6-gingerol in reducing the generation of proinflammatory mediators such as nitric oxide and prostaglandin E2 in mouse macrophages [244]. The anti-inflammatory mechanisms of ginger are associated with an enhancement in anti-inflammatory cytokines and a decline in proinflammatory cytokines. Ginger can lower levels of inflammatory markers like TNF-α and IL-8 in the lungs, which helps improve airflow (Table 8) [245,246,247].

Ginger may efficiently reduce lung damage and protect the lungs from severe damage due to hyperoxia and inflammation. Studies showed that ginger may be an alternative option for the treatment of Bronchopulmonary dysplasia [248].

A randomized controlled trial evaluated the efficacy and safety of ginger on clinical and paraclinical features in outpatients with COVID-19. The outpatients with confirmed COVID-19 were randomly assigned in a 1:1 ratio to receive ginger (1000 mg 3 times a day for 7 days) or a placebo. The trial found no significant improvement in viral clearance or differences in oxygen saturation, body temperature, or respiratory rate between the groups. However, by the seventh day, the ginger group showed a significant reduction in pulmonary infiltrates. Ginger did not significantly impact clinical parameters but was found to be safe and effective in reducing pulmonary infiltrates [109].

Additionally, ginger also has antioxidant properties that help fight oxidative stress, which is common in long-term lung diseases. It works by removing harmful free radicals and improving the body’s natural ability to protect itself from damage. This supports lung health and helps prevent tissue damage [249].

Ginger can help reduce breathlessness and improve the quality of life for COPD patients by decreasing airway inflammation. Ginger supplementation has been found to improve oxidative stress markers, such as increased superoxide dismutase, catalase, and glutathione, which are linked to the progression of COPD [250,251].

A study assessed the effects of ginger extract on delayed gastric emptying, ventilator-associated pneumonia, and clinical outcomes in patients with adult respiratory distress syndrome (ARDS). The trial included thirty-two ARDS patients reliant on mechanical ventilation and nasogastric feeding. They were randomized into a placebo group and another group that received 120 mg of ginger extract. The results showed that the ginger group tolerated more feeding in the first 48 h compared to the control group. There was a trend toward a decrease in pneumonia in the ginger group. The reported intensive care unit (ICU) mortality showed no significant difference between groups. However, the ginger group had more ventilator-free days and ICU-free days. The trial indicated that ginger extract may help reduce delayed gastric emptying and the incidence of ventilator-associated pneumonia in ARDS patients [112].

Studies indicate that ginger can reduce bronchial inflammation and mucus production, providing relief for people with chronic bronchitis. It supports the body’s natural ability to clear mucus from the airways, allowing patients to cough up mucus more easily [237,245].

Ginger is an essential ingredient in traditional medicine, renowned for its powerful health benefits. All nine randomized controlled trials (Table 1) investigating *Zingiber officinale*’s immune support potential demonstrated that Ginger can significantly impact RTIs, yielding evidence that ranges from moderate to strong.

**Table 8:** Properties of *Zingiber officinale* relevant to Respiratory Tract Infections.

Plant	Properties	Contribution	References
*Zingiber officinale*	Immunomodulatory Properties	Stimulates cytokine production (IL-1, IL-8, TNF-α)	[245,246,247]
	Antiviral activity	Supportive role in mild RTIs	[238,239]
	Antibacterial activity	Supportive role in mild RTIs	[252]
	Anti-inflammatory effects	Limits the production of phoshatidylinositol-3-kinase, protein kinase B, and NF-κB	[244]
	Antitussive effect	Lowers sensitivity of the cough reflex and alleviates throat irritation	[237]

### 4.8. Curcuma longa

**Figure 9:**
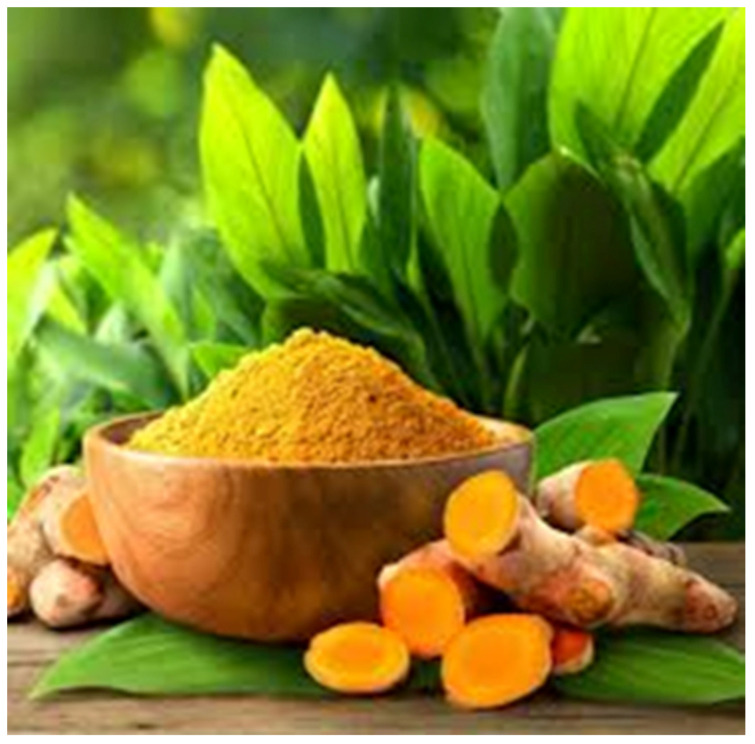
*Curcuma longa*.

*Curcuma longa (C. longa)*, commonly known as Turmeric, is a flowering plant in the ginger family *Zingiberaceae* (Figure 9). The rhizomes can be used fresh, boiled in water and dried, after which they are ground into a deep orange-yellow shelf-stable spice powder [253,254].

*C. longa* contains bioactive compounds that have anti-inflammatory, antioxidant, antimicrobial, as well as anti-diabetic, antitumor, and neuroprotective properties [255].

Turmeric is rich in a variety of active pharmaceutical ingredients, including diphenylalkanoids, terpenoids, aromatics, steroids, and fatty acids. Additionally, turmeric also contains a variety of macro and micro elements, including K, Mg, Ca, Na, Al, Cr, Cu, Mn, Rb, Sr, and Zn [256].

*C. longa* showed both anti-bacterial and anti-viral activity. Study reported Minimal inhibitory concentrations (MIC) values within the range of 125–1000 μg/mL against *Bacillus subtilis*, *Escherichia coli*, *Pseudomonas aeruginosa*, *Staphylococcus aureus*, and *Vibrio cholerae* [257].

Another study [258] evaluated the antimicrobial effects of turmeric against three Gram-negative (*E. coli*, *K. pneumoniae*, *Pseudomonas* sp.) and three Gram-positive (*E. faecalis*, *L. innocua*, *S. aureus*) bacteria. *E. coli* and *K. pneumoniae* demonstrated tolerance to turmeric concentrations of 0.1 mg/mL and 1 mg/mL. The growth of *Pseudomonas* was slowed at the lowest concentration tested and significantly inhibited at the medium concentration. In contrast, the Gram-positive bacteria exhibited different growth patterns and appeared to be more sensitive to turmeric. The growth curves for *S. aureus* in media with all tested turmeric concentrations showed significant suppression compared to the control group. As a result, turmeric concentrations ranging from 0.1 mg/mL to 1 mg/mL displayed at least some antimicrobial activity against all Gram-positive bacteria tested. This activity ranged from complete inhibition (as seen with *S. aureus*) to limiting the growth kinetics of other bacteria such as *E. faecalis* and *L. innocua* [258].

Three curcuminoid compounds, curcumin, demethoxycurcumin and bisdemethoxycurcumin, could serve as potential supplementary agents in preventing and treating diseases caused by influenza viruses [259]. These compounds exhibited inhibitory activity against novel *Influenza* strains, including H9N2, H1N1, and the oseltamivir-resistant novel H1N1 (H274Y mutant) (Table 9) [260].

Other trials are investigating the effects of curcumin on COVID-19 patients. A double-blind, randomized clinical trial explored nanocurcumin’s effect on the clinical manifestations of patients hospitalized with mild-to-moderate COVID-19. All patients received standard coronavirus treatment, and in addition, four times a day for two weeks, the curcumin group received 40 mg of nanocurcumin, while the control group received a placebo. Compared with the placebo, nanocurcumin minimized coughs, fatigue, myalgia, oxygen demand, oxygen usage, and respiratory rate. By the time of discharge, the curcumin group exhibited a significantly greater increase in blood oxygen saturation (SPO_2_) than the control group [117].

The anti-inflammatory activity of curcumin is focused on significantly inhibiting the increased levels of key pro-inflammatory mediators such as TNF-α, IL-1β, matrix metalloproteinases (MMP): MMP-1, and MMP-3 via the mTOR pathway [261]. The transcription factor NF-κB, as a central regulator of inflammatory responses, plays a crucial role in the pathogenesis of diverse inflammatory disorders. Curcumin possessed an anti-inflammatory effect via blocking the NF-κB signalling pathway [262].

Reanalysis of data from two randomized, double-blind, placebo-controlled trials revealed the results of the effect of *C. longa* extracts on serum glucose levels in the presence of low-grade inflammation. Overweight participants aged 50 to 69 years were analyzed based on the inflammatory marker high-sensitivity C-reactive protein (hsCRP). Participants consumed either a hot water extract of *C. longa* or a placebo for 12 weeks, during which we measured their serum hsCRP and fasting serum glucose levels. The mean baseline hsCRP value was used to stratify participants into two subgroups: a low-hsCRP subgroup and a high-hsCRP subgroup. In the low-hsCRP subgroup, the study disclosed no significant difference in fasting serum glucose levels between the two groups, but in the high-hsCRP subgroup, the *C. longa* extract group had significantly lower levels of serum hsCRP and fasting serum glucose than the placebo group. The study indicates a hot water extract of *C. longa* may help to improve systemic glucose metabolism in people with chronic low-grade inflammation [120].

The essential oils extracted from the rhizome of turmeric also have anti-inflammatory activity. α-turmerone, ar-turmerone, and β-turmerone were the main components in essential oils, accounting for 12.9%, 42.6%, and 16.0%, respectively [263]. Aromatic Turmerone, a turmeric oil derived from turmeric, exhibited anti-inflammatory activity against NF-κB and STAT3 pathways [264,265].

A randomized controlled trial investigated the effects of a curcuminoid-piperine combination on systemic oxidative stress and clinical symptoms in patients with chronic pulmonary issues due to sulfur mustard exposure and suggests that curcuminoids may be a safe and effective adjunct treatment for these patients. Eighty-nine participants were randomly assigned to either the active treatment group (n = 45) or a placebo group (n = 44) for 4 weeks. High-resolution computed tomography confirmed bronchiolitis obliterans in all subjects. Effectiveness was measured by changes in serum levels of reduced glutathione (GSH) and malondialdehyde (MDA), as well as symptom severity. At the trial’s end, the curcuminoid-piperine combination had a significantly greater impact on elevating GSH, reducing MDA and improving the Health-Related Quality of life [118].

All of the listed properties of turmeric and the clinical studies conducted support the fact that it can be used successfully in both acute and chronic respiratory conditions. Every clinical trial we found was a randomized controlled CT, ensuring the highest standard of evidence.

**Table 9:** Properties of *Curcuma longa* relevant to Respiratory Tract Infections.

Plant	Properties	Contribution	References
*Curcuma longa*	Antiviral activity	Supportive role in mild RTIs	[259,260]
	Antibacterial activity	Moderate activity in RTIs	[258]
	Anti-inflammatory effects	Inhibits pro-inflammatory cytokines (TNF-α, IL-1, IL-6), NF-κB signalling	[261,264,265]
	Antioxidant	Protection from oxidative stress during infection	[255]

In our review for the examined study period (2000–2025), we identified five clinical trials that matched our criteria for respiratory tract infections. All trials presented in Table 1 were well-structured, being randomized, double- or triple-blinded, controlled or combined randomized and controlled designs. This contributed to consistent and reliable results across the studies.

The discussed natural agents may offer supportive benefits in the management of URTIs and LRTIs by alleviating symptoms, supporting immune function, and improving comfort. Overall, the strength of evidence varies considerably across products, study designs, and clinical endpoints, limiting the ability to draw definitive conclusions regarding efficacy.

The evidence suggests differential utility depending on the anatomical site of infection. Products with demulcent and antitussive properties, like *Althaea officinalis*, were more frequently evaluated in URTIs, where symptom relief rather than pathogen eradication is the primary therapeutic goal. Conversely, expectorant and bronchospasm-relieving agents such as *Hedera helix* and *Thymus vulgaris* were more commonly studied in LRTIs such as acute bronchitis.

Among the reviewed interventions, *Echinacea* spp., *Pelargonium sidoides*, *Hedera helix*, *Thymus vulgaris*, and *Sambucus nigra* were supported by the most consistent clinical evidence, including randomized controlled trials conducted. These products were primarily associated with reductions in cough severity, symptom duration, and overall disease burden, particularly in acute bronchitis and viral upper respiratory tract infections.

In contrast, other commonly used plants, such as marshmallow root, plantain, licorice root, chamomile, fennel, and anise, were supported largely by traditional use, observational studies, or small clinical trials. While these products are frequently incorporated into different formulations and may provide symptomatic relief, the current evidence base remains limited, and their effects cannot be reliably quantified.

It is noteworthy that the majority of patients who use herbal products/dietary supplements do not inform their doctors about this. The healthcare professionals should collect more information about the supplements being taken, the duration of their intake, and whether there is a response that the patient would note as a significant improvement. They should also actively engage with patients to discuss their use of herbal products and to provide counselling on this subject to prevent possible adverse effects and herb-drug interactions.

Clinical trials investigating plant-derived agents often encounter a challenge due to heterogeneity in formulation. Compared to the single-molecule pharmaceuticals, plant adjuncts are complex mixtures whose composition can vary widely depending on multiple factors.

The products consist of various plant species and subspecies, and many pharmaceutical formulations include a mixture of different plant-derived components.

The geographic origin, selection of the most suitable alternative cultivation area, and cultivation conditions are crucial factors. Additionally, the timing of harvesting and post-harvest processing plays a significant role. Various studies may utilize different extraction methods, such as aqueous, ethanolic, or supercritical extractions, for the isolation of the bioactive compounds of the plants.

In many cases, the pharmaceutical formulations depend on the benefits and effectiveness of treatments derived from different parts of plants: roots, leaves, and seeds.

Various clinical trials investigate different dosage forms such as capsules, tinctures, sprays, teas and standardized extracts. Even when the same plant is studied, variability in the concentration of active compounds can lead to substantially different biological effects.

This heterogeneity complicates the interpretation and comparison of clinical trial results. It becomes difficult to determine whether observed effects are attributable to the plant itself, a specific active constituent, or differences in formulation and bioavailability.

Plant-derived compounds frequently demonstrate significant variability in their absorption and metabolism. This variability can be influenced not only by the specific formulation of the compounds, but also by the presence of other co-existing substances within the extract. The efficiency with which these compounds are absorbed in the body can vary based on how they are processed, their chemical structure, and the presence of other ingredients that may enhance or inhibit their effects. This inconsistency may result in varied therapeutic outcomes across different studies, populations, and individual responses.

Also, we acknowledge that blinding and placebo control can be more difficult to achieve in trials involving plant-based products, when they have distinctive tastes, colours, or odours. This may increase the risk of performance and detection bias.

The **Limitations of the study** include a lack of definitive conclusions of evidence for the discussed plant adjuncts due to the heterogeneity of formulations, dose consistency and bioavailability, which limits comparability across studies. Without rigorous standardization and transparent reporting, drawing firm conclusions about efficacy and safety in diverse patient groups remains challenging.

## 5. Conclusions

The discussed plant-derived agents exhibit expectorant, anti-inflammatory, antimicrobial, antiviral and antioxidant activities that complement conventional therapy. Their use may enhance patient’s overall comfort and potentially reduce the duration or severity of symptoms when used appropriately. To provide more robust evidence for the efficacy in patients with RTIs, larger controlled clinical trials would be welcomed, though the challenges associated with such research must be acknowledged.

## Figures and Tables

**Table 2:** Properties of *Echinacea* spp. relevant to Respiratory Tract Infections.

Plant	Properties	Contribution	References
*Echinacea* spp.	Immunomodulatory properties	Enhances activation of macrophages, dendritic cells, phagocytic activity and antigen presentation	[125,126]
	Antiviral activity	Mostly in viral URTIs	[130,131]
	Anti-inflammatory effects	Reduced inflammatory response	[128]
	Antioxidant properties	Protection of respiratory epithelial cells from oxidative damage, supports mucosal barrier integrity	[133,134,135]
	Mucosal immunity effects	Supports local immune response and increases secretory IgA levels	[39]

**Table 3:** Properties of *Pelargonium sidoides* relevant to Respiratory Tract Infections.

Plant	Properties	Contribution	References
*Pelargonium sidoides*	Immunomodulatory properties	Enhances activation of macrophages, phagocytic activity, cytokine production	[154]
	Antibacterial properties	Inhibits adhesion of bacteria to respiratory epithelial cells. May limit early pathogen colonization and spread	[64]
	Antiviral activity	Mostly in viral URTIs	[152,153]
	Anti-inflammatory effects	Reducing production of pro- inflammatory mediators Limiting airway epithelial damage	[155]
	Anti-adhesive and Mucokinetic effects	Reduces bacterial attachment to mucosal surfaces Enhances ciliary beat frequency and mucociliary clearance	[64]

**Table 7:** Properties of *Sambucus nigra* relevant to Respiratory Tract Infections.

Plant	Properties	Contribution	References
*Sambucus nigra*	Immunomodulatory Properties	Stimulates cytokine production (IL-1, IL-6, TNF-α)	[227]
	Antiviral activity	Inhibits viral entry and replication	[219,220]
	Anti-inflammatory effects	Reduces pro-inflammatory mediator expression	[225,226]
	Mucus clearance	Supports epithelial barrier with antioxidant and anti-inflammatory effects	[225,226]

## Data Availability

Data are contained within the review.
